# Relationship between the microenvironment and survival in kidney transplantation: a bibliometric analysis from 2013 to 2023

**DOI:** 10.3389/fimmu.2024.1379742

**Published:** 2024-03-26

**Authors:** Chun-Lian Huang, Xin-Yu Fu, Yi Feng, Xiao-Kang Li, Yi Sun, Xin-Li Mao, Shao-Wei Li

**Affiliations:** ^1^ Department of Infectious Diseases, Taizhou Hospital of Zhejiang Province Affiliated to Wenzhou Medical University, Linhai, Zhejiang, China; ^2^ Taizhou Hospital of Zhejiang Province affiliated to Wenzhou Medical University, Linhai, Zhejiang, China; ^3^ Key Laboratory of Minimally Invasive Techniques and Rapid Rehabilitation of Digestive System Tumor of Zhejiang Province, Linhai, Zhejiang, China; ^4^ Division of Transplantation Immunology, National Research Institute for Child Health and Development, Tokyo, Japan; ^5^ MRL Global Medical Affairs, MSD China, Shanghai, China; ^6^ Department of Gastroenterology, Taizhou Hospital of Zhejiang Province affiliated to Wenzhou Medical University, Linhai, Zhejiang, China; ^7^ Institute of Digestive Disease, Taizhou Hospital of Zhejiang Province Affiliated to Wenzhou Medical University, Linhai, China

**Keywords:** CiteSpace, bibliometrics, VOSview, kidney transplantation, immune microenvironment, survival

## Abstract

**Background:**

Kidney transplantation is considered the most effective treatment for end-stage renal failure. Recent studies have shown that the significance of the immune microenvironment after kidney transplantation in determining prognosis of patients. Therefore, this study aimed to conduct a bibliometric analysis to provide an overview of the knowledge structure and research trends regarding the immune microenvironment and survival in kidney transplantation.

**Methods:**

Our search included relevant publications from 2013 to 2023 retrieved from the Web of Science core repository and finally included 865 articles. To perform the bibliometric analysis, we utilized tools such as VOSviewer, CiteSpace, and the R package “bibliometrix”. The analysis focused on various aspects, including country, author, year, topic, reference, and keyword clustering.

**Results:**

Based on the inclusion criteria, a total of 865 articles were found, with a trend of steady increase. China and the United States were the countries with the most publications. Nanjing Medical University was the most productive institution. High-frequency keywords were clustered into 6 areas, including kidney transplantation, transforming growth factor β, macrophage, antibody-mediated rejection, necrosis factor alpha, and dysfunction. Antibody mediated rejection (2019-2023) was the main area of research in recent years.

**Conclusion:**

This groundbreaking bibliometric study comprehensively summarizes the research trends and advances related to the immune microenvironment and survival after kidney transplantation. It identifies recent frontiers of research and highlights promising directions for future studies, potentially offering fresh perspectives to scholars in the field.

## 1 Introduction

Kidney transplantation has become the preferred procedure for the treatment of patients with kidney failure because it is the most effective treatment, both medically and economically (1–3). Advances in immunosuppressive drugs and protocols have markedly reduced the incidence of graft rejection and improved survival rates of patients in recent years (3–5). Nonetheless, improving long-term transplant outcomes remains a crucial challenge (4). Many current studies have shown that allograft reaction is the major cause of late kidney transplant failure (6–8). Therefore, new treatments are necessary to improve long-term graft survival and suppress allograft reactions.

Many studies have identified that the immune microenvironment (immune cells, cytokines, etc.) plays a key role in coordinating the immune response after kidney transplantation. Therefore, they can be investigated for potential applications in new therapeutic strategies (9–11). Cells in the immune microenvironment play a critical role in prolonging the survival of kidney transplant patients after kidney transplantation (12, 13). Therefore, this study systematically explores the publications and hotspots of research related to the relationship between the immune microenvironment and patient survival after kidney transplantation.

The term “bibliometric analysis” refers to the use of mathematical and statistical methods that are commonly used to provide a comprehensive picture of the current status of a field, publication trends, scientific output of researchers, institutions, and countries, and future research hotspots (14, 15). This method has been widely used in several fields. To the best of our knowledge, there have been no published bibliometric analyses of the immune microenvironment after kidney transplantation. Therefore, this study is aimed to reveal difficult problems and research hotspots related to the immune microenvironment after kidney transplantation over the past 10 years.

## 2 Materials and methods

### 2.1 Data collection

**Figure 1:**
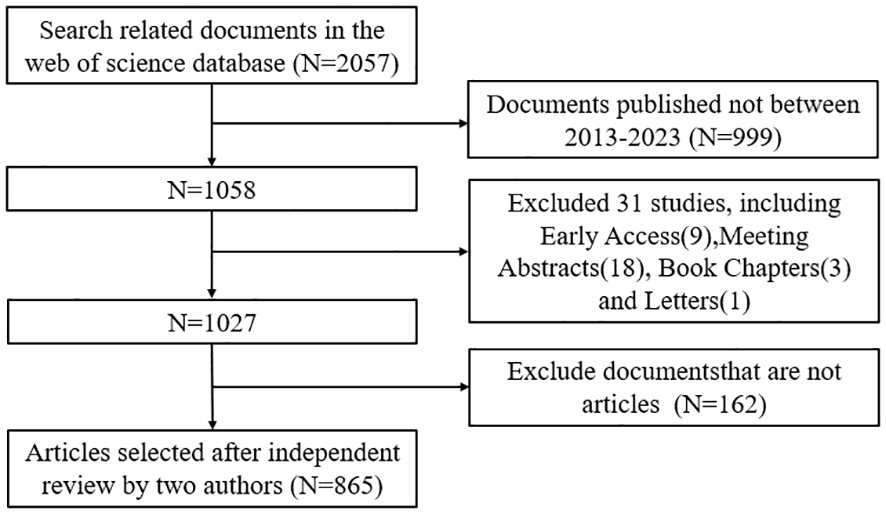
Screening flowchart for inclusion of studies.

We conducted a literature search on the Web of Science Core Collection (WoSCC) database on August 21. The search formula was as follow TS = (“Cytokines” OR “Chemokines” OR “Growth Differentiation Factor 15” OR “Hematopoietic Cell Growth Factors” OR “Hepatocyte Growth Factor” OR “Interferons” OR “Interleukin 1 Receptor Antagonist Protein” OR “Interleukins” OR “Leukemia Inhibitory Factor” OR “Lymphokines” OR “Monokines” OR “Oncostatin M” OR “Osteopontin” OR “Thymic Stromal Lymphopoietin” OR “Transforming Growth Factor beta” OR “Tumor Necrosis Factors”) AND (“Kidney Transplantation”)AND (“Language = English”), and the type of documents is set to “articles”. The search was limited to the period from August 21, 2013 to August 21, 2024. Two authors read the abstract and full text and exclude articles that are not relevant to the articles. The flow chart of the included articles is shown in **Figure 1**, and a total of 865 articles were selected for bibliometric analysis.

### 2.2 Data analysis

In this study, CiteSpace 6.1. R3, VOSviewer 1.6.18, and Microsoft Excel 2019 were used for the bibliometric analysis, visualization methods, and integration analysis (5, 16). VOSviewer can extract key information from a wide range of publications, including lead authors, and analyze country and institution, keywords, scientific partnerships, citations, and co-citations (17). CiteSpace explores the current state of research, research hotspots, research frontiers, and development process of a scientific field by generating a series of visual knowledge maps that reveal the trends in the field (18). The Excel software program was used to analyze the annual publications.

## 3 Results

### 3.1 Annual publications

**Figure 2:**
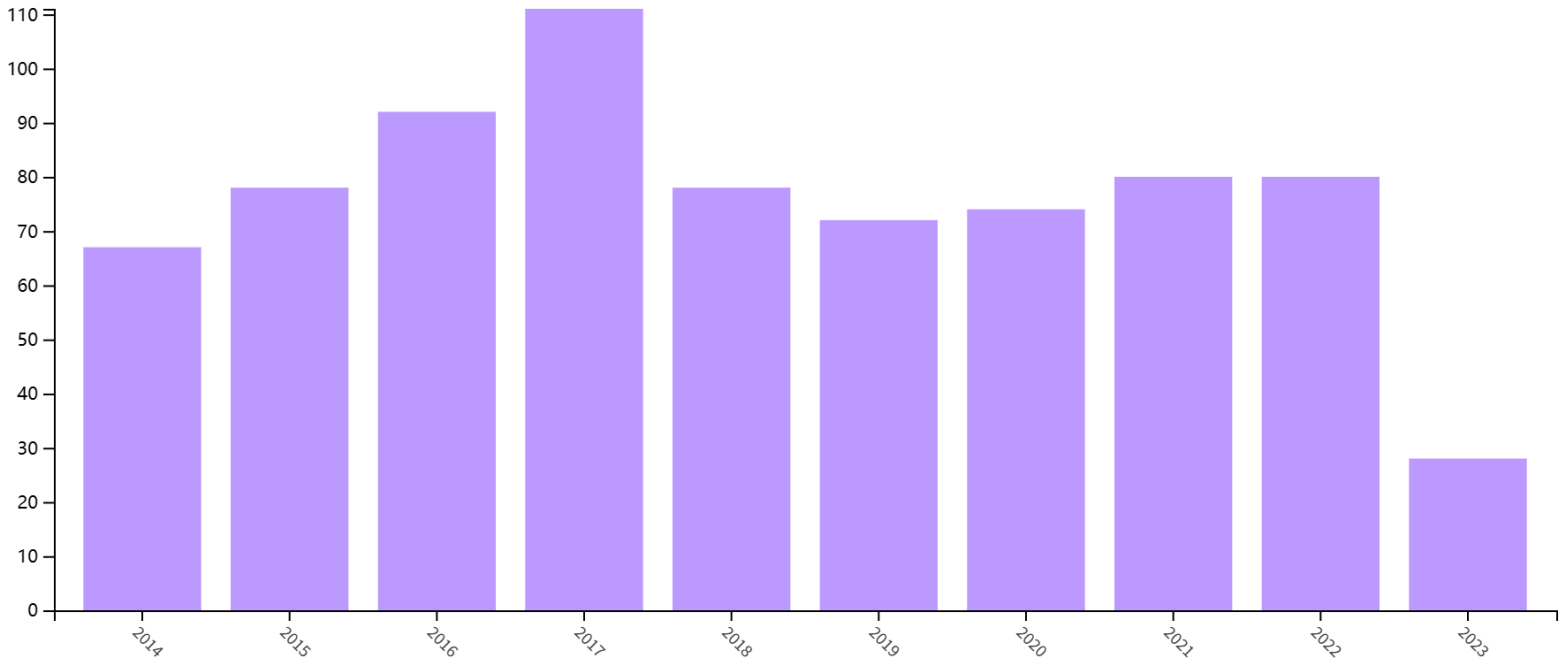
Annual yield of research on the immune microenvironment related to survival after kidney transplantation.

Based on our inclusion criteria (**Figure 1**), a total of 865 articles were included in the study. As shown in **Figure 2**, the number of papers related to the immune microenvironment after kidney transplantation has fluctuated over the past 10 years, reaching a peak in 2017, with a generally stable trend.

### 3.2 Distribution of countries/regions and institution

**Figure 3:**
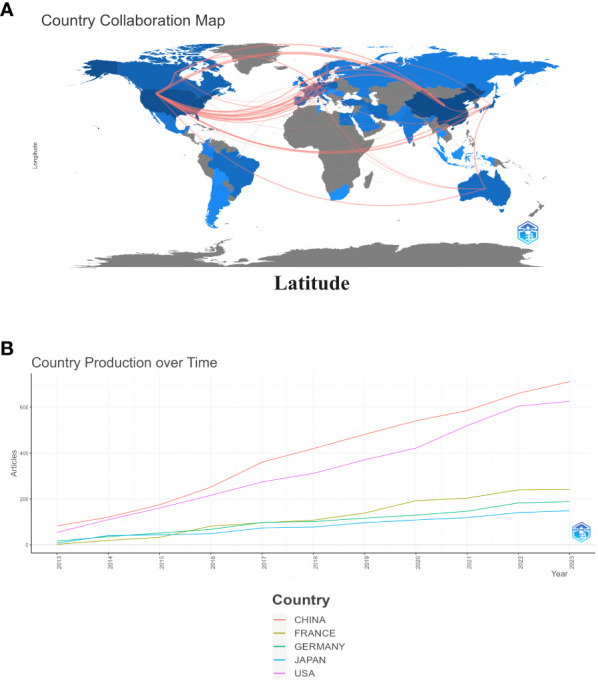
Map of collaboration between different countries **(A)**. Trends in publication distribution in the top five countries **(B)**.

**Figure 4:**
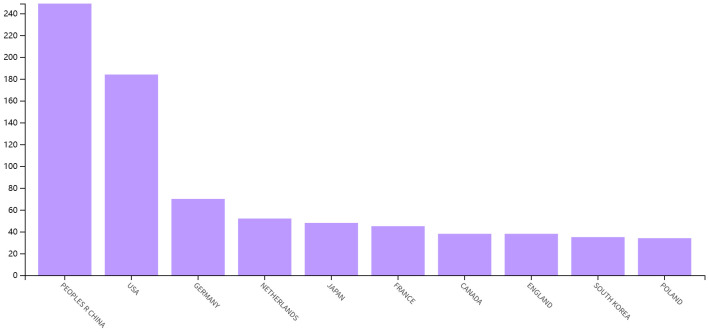
Top 10 countries in terms of articles published in the field between 2013-2023.

As shown in **Figure 3A**, China is the most published country, followed by the United States, Germany, Netherlands and Japan. Afterward, we filtered and visualized all countries based on the number of publications greater than or equal to 2, and built a collaboration network (**Figure 3B**). We discovered that there are many positive collaborations between different countries. For example, China has close collaborations with the United States; the United States has also actively collaborated with Australia, Japan, France, and the United Kingdom. As shown in the figure, the top six universities come from five countries, with one-third of them located in China. The six universities that have published the most relevant papers are Harvard Medical School, Leiden University, Nanjing Medical University, Oslo University Hospital, Pomeranian Medical University, and Sichuan University. In the last decade, the number of papers published in China has increased rapidly year by year, followed by France (**Figure 4**).

### 3.3 Authors and institutions of relevant articles

**Figure 5:**
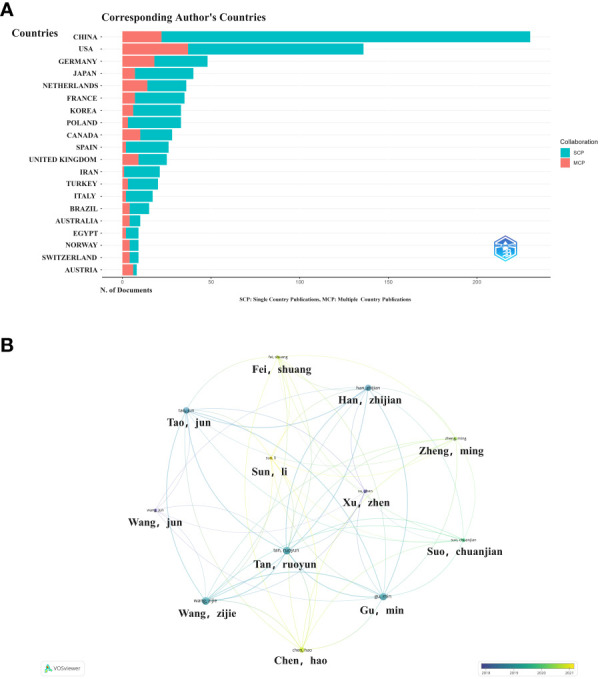
Collaborative networks between authors and between countries **(A)**. Countries associated with authorsVisualization map between authors **(B)**.

Among these publications, the Chinese authors published the most papers, followed by the United States (**Figure 5A**). We constructed a collaborative network based on authors with a number of publications greater than or equal to two. The largest nodes and the most relevant publications, and they were closely related to each other (**Figure 5B**).

### 3.4 Analysis of co-cited references and reference burst

**Figure 6:**
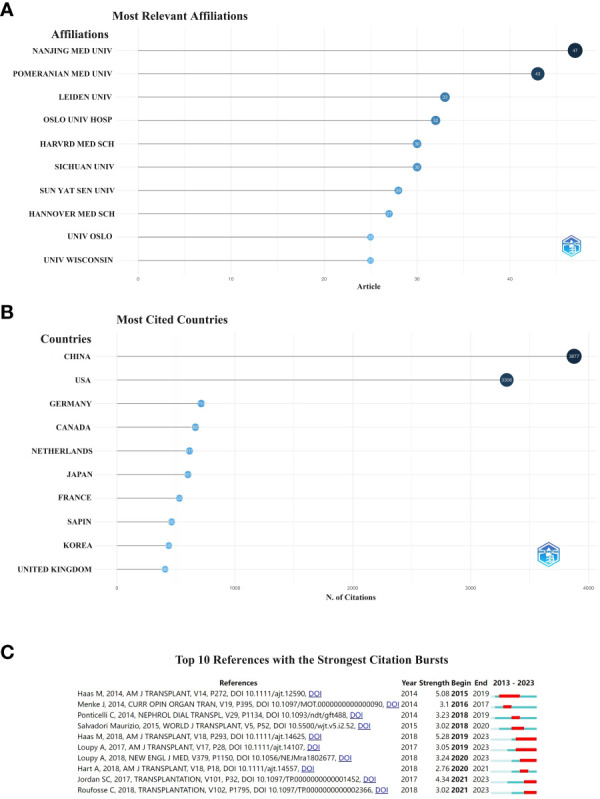
Institution **(A)**, country **(B)** and the 10 most cited references to which the publication relates. The red bar indicates the year with the most citations **(C)**.

When two or more references are cited in more than one article, the two references are considered to be in a co-citation relationship (18). The most cited country was China with 3,877 citations, followed by the United States with 3,306 citations (**Figure 6A**). The main cited relevant institution was Nanjing Medical University with 47 articles, followed by Pomeranian Medical University with 43 articles (**Figure 6B**).

A citation burst is a document that is frequently cited by scholars in a particular field over a certain period of time. In our study, CiteSpace identified ten documents with strong citation bursts (**Figure 6C**). The earliest citation bursts for references appeared in 2015 and the latest in 2021. The literature with the strongest citation outbreak (strength = 5.28) is “The Banff 2017 Kidney Meeting Report: Revised diagnostic criteria for chronic active T cell-mediated rejection, antibody-mediated rejection, and prospects for integrative endpoints for next-generation clinical trials” (19), citing an outbreak period of 2019–2023. Overall, the outbreak strength of the ten publicatio.

### 3.5 Keywords used in co-citation networks

**Figure 7:**
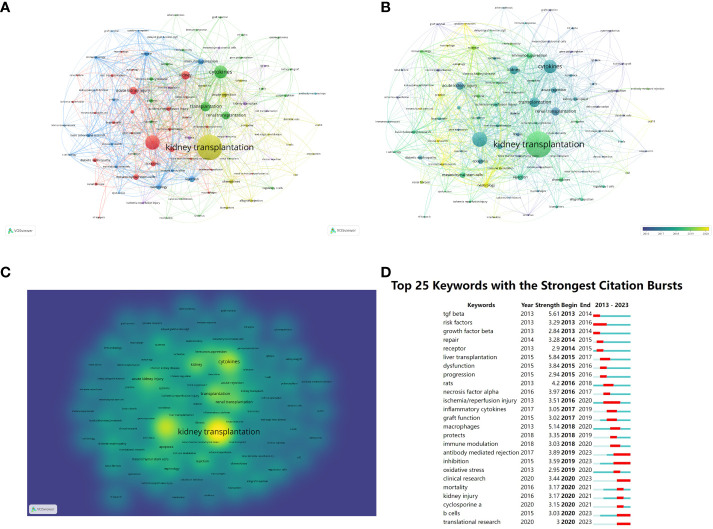
Clustering of keywords in studies related to the immune microenvironment and survival after kidney transplantation **(A–C)**. Top 25 most cited keywords **(D)**.

Different visual clusters of keywords used in published articles were mapped using VOSview and CiteSpace (20). Clustered network visualizations and frequency heat maps of keywords were created on VOSview. CiteSpace was connected to the carrot 2 system to analyze the key topics and related common words, which were shown as follows: kidney transplantation, cytokines, rapid kidney injury, mesenchymal stem cells, and immunosuppression (**Figures 7A–C**). The yellower color represents the latest hot keywords. We used CiteSpace software to complete the analysis of keyword bursts in the immune microenvironment of kidney transplantation (**Figure 7D**). “TGF-β”, “macrophage” and “antibody-mediated rejection” appeared earlier and were noticed earlier. The keywords with the strongest cited outbreaks were even transplantation (strength=5.84), TGF-β (strength=5.61) and macrophages (strength=5.14). Macrophage is the keyword with stronger outbreaks that appeared in 2018, which could be a hotspot for research or a turning point with prospective research implication.

## 4 Discussion

### 4.1 General information study

This is the first bibliometric and visual analysis of the immune microenvironment in kidney transplantation between 2013 and 2023. A total of 865 articles from SCI-E were included in this study, and each retrieved article was screened to ensure relevance to the topic. The publications and citation frequency related to the immune microenvironment after kidney transplantation have shown a consistent increase, making it an active research topic over the last decade (**Figure 2**). China, the United States, and Germany were major contributors to this research area. China published the most cited papers, indicating that it has conducted in-depth research in this area (**Figure 3**). Among the top six selected institutions, the United States institutions mainly collaborated with German research institutions.

China and the United States are the main countries conducting research on the immune microenvironment of kidney transplantation, with China in the first place. About one-third of the top 6 research organizations are located in China, followed by the United States. We have noticed close cooperation between the four countries - the United States, China, Germany and Japan. In terms of authors, there are good collaborations between some authors, such as Ruoyun Tan, Li Sun, Zhen Xu, Min Gu, and Zijie Wang. One of the most influential is the article published in Frontiers in immunology in 2021 and 2022. It is entitled “Combined Immunotherapy With Belatacept and BTLA Overexpression Attenuates Acute Rejection Following Kidney Transplantation” and “Diagnostic Biomarkers and Immune Infiltration in Patients With T Cell-Mediated Rejection After Kidney Transplantation.” They focused on the role played by T-lymphocytes in the immune microenvironment after kidney transplantation in mediating transplant rejection and its clinical use (13, 21).

In terms of institutions, we find that Nanjing Medical University has the most publications. The authors, Ruoyun Tan, Li Sun Min Gu, and Zijie Wang, are from Nanjing Medical University. China and the United States as major countries for research, but the breadth and strength of inter-institutional collaborations are not ideal. Clearly, this situation will hinder the development of the research field in the long run. Therefore, we strongly recommend that research institutions in various countries develop extensive cooperation and communication to promote the development of the immune microenvironment in kidney transplantation.

### 4.2 Hotspots and Frontiers

The basic structure of research in the field of the immune microenvironment after kidney transplantation can be revealed using literature co-citation networks and keyword clustering analyses. The strongest references citation bursts were the meeting summaries of the 12th and 13th Banff Transplant Pathology Conferences. The goal of both meetings hopes to provide a greater understanding of graft immune rejection through the continued integration of advances in histologic, serologic, and molecular diagnostic techniques. To provide precise comprehensive scoring, accurate and routine diagnostics for clinical trials (22, 23). By scrutinizing these analyses, a great deal of valuable information can be gleaned, including TGF-β, and macrophages and antibody-mediated rejection. These findings help to identify emerging trends and research hotspots in the field of the immune microenvironment after kidney transplantation.

#### 4.2.1 Kidney transplantation and macrophages

Macrophages are a key immune system for innate immunity and have a wide range of tissue-resident cell surface receptors, including pattern recognition receptors for damage-associated molecular patterns (DAMPs), complement products, chemokines, Fc fragments, and toll-like receptors (TLRs) (17). It is well known that macrophages play a key role in organogenesis, tissue homeostasis and promotion of tissue injury.

A unique feature of macrophages in allogeneic transplantation is that donor macrophages are transferred with the donor organ at the time of transplantation and recipient monocyte-derived macrophages are subsequently recruited into the allogeneic graft (18). In early severe renal rejection transplants, macrophages account for approximately 60% of the immune cells. Macrophages play a key role in acute cell-mediated rejection and antibody-mediated rejection (24, 25). The major cause of long-term renal transplant failure is histologic interstitial fibrosis and tubular atrophy. The current study found that intercellular communication between renal parenchymal cells and donor-derived macrophages, detected several years after transplantation, plays a key role in the proliferation of damage (19, 20).

In summary, there is growing evidence of the important role of macrophages in tissue inflammation and repair. In recent years, there has been a renewed and increasing emphasis on macrophages. Thus, macrophages are promising therapeutic targets for clinical transplantation (26). Currently, regulatory cell therapy, which aims to protect the immunomodulation of organ grafts, has become an attractive therapeutic approach (27). This approach focuses on expanding specific populations of regulatory immune cells *in vitro* in the form of cell-based medicinal products (CBMPs), which are then infused into transplant recipients to minimize graft rejection. The CBMPs studied so far mainly consist of two polyclonal T regulatory (pTreg-1 and pTreg-2), two donor antigen-reactive Treg (darTreg-CSB and darTreg-sBC), a tolerogenic dendritic cell (autologous tolerogenic dendritic cell [ATDC]) and a regulatory macrophage (Mreg) cell product (28). The current study found that compared to immunosuppressants, regulatory cell therapy has good efficacy in both early and late kidney transplantation with fewer infectious complications and side effects. This is serving as a major direction for future research (27–32).

#### 4.2.2 Kidney transplantation and TGF-β

Cytokines play a key role in coordinating the immune response after kidney transplantation. Therefore, it is crucial to understand the role of cytokines in the allogeneic immune response (33). Among all cytokines, TGF-β is a multifaceted cytokine that regulates pro- and anti-inflammatory responses depending on the microenvironment and target cell type (34). In addition, TGF-β signaling regulates a broad spectrum of biological processes involved in tissue homeostasis and injury responses, including cell growth and differentiation, migration, survival and death (35). To date, three major isoforms of TGF-β (TGF-β1, TGF-β2 and TGF-β3) encoding for TGFB1, TGFB2 and TGFB3, respectively, have been identified in humans. Of these, TGF-β1 is the most common and best characterized isoform (10).

Interstitial fibrosis is an important factor in graft loss in chronic transplant kidney injury (19). TGF-β1 is a key fibrotic cytokine involved in fibrosis in a variety of chronic kidney and other organ diseases (20). Expression of TGF-β can be detected in allograft patients, especially in failed kidney graft tissues (36).

In kidney transplantation, TGF-β1 has been a topic of interest and most investigators believe that TGF-β1 affects allograft survival in different ways (37). It has been shown that TGF-β1 cells are closely associated with the short-term prognosis of clinical kidney transplantation. Several clinical studies have found that elevated serum TGF-β1 levels after long-term kidney transplantation may have a positive effect on long-term graft survival and may be a predictor of graft survival and function (21, 24–26).

### 4.3 Kidney transplantation and cell therapy

The combination of general immunosuppressive drugs improves graft survival cycles. However, graft survival has been shortened by chronic rejection and immunosuppressive side effects and has been stagnant for the past decade (1, 38). To address this problem, organ transplantation urgently requires new strategies to reduce our dependence on immunosuppressive drugs to prevent allograft rejection. Currently, the use of cell-based drug products is the state-of-the-art method to reduce immunosuppression in organ transplantation (28). Regulatory cell therapy has emerged as an attractive therapeutic approach to establish immunomodulation aimed at protecting organ grafts (39–41). Currently, common types of regulatory cell therapy include regulatory T cells (Treg), monocyte-derived dendritic cells, and regulatory macrophages. Of these, regulatory T cells are most commonly utilized in clinical practice (42–44). Other cell therapies are currently in clinical testing (45, 46). Current studies have shown that cell therapy is safe and has fewer infectious complications. Thus, immune cell therapy is a potentially useful treatment for renal transplant recipients, reducing the burden of general immunosuppression as well as improving long-term outcomes (28, 47).

### 4.4 Advantages and shortcomings

This study has several unique advantages over traditional reviews. First, we systematically analyzed the studies on the correlation between immune microenvironment and survival after kidney transplantation for the first time by using bibliometric methods. Second, the bibliometric analysis objectively and comprehensively quantifies and evolves the research hotspots and trends in a certain field through mathematical techniques, which can provide a comprehensive guide for scholars concerned with related research. Finally, in this review, not only the evidence of hotspots and trends of the correlation between immune microenvironment and survival after kidney transplantation is objectively presented, but also the current research results and outlook are systematically summarized. Therefore, it is hoped that the summarization of the existing research results will help researchers to quickly identify their strengths and weaknesses, thus promoting the development of the field.

Of course, but there are still some limitations that may affect its findings. First, the data used in this paper are exclusively from the WoSCC database, excluding other databases, which may have missed some relevant studies. Second, we only analyzed literature published in English, ignoring studies in other languages. Although the search terms related to the immune microenvironment contained most of the content, they were still lacking, leading to potentially biased results. Some of the relevant literature was not included in the study. The year 2023 is not yet finished ending and only currently published literature was included, which may have excluded some valuable information. Finally, only articles were included without considering political and social publications such as reviews, editorials and books.

## 5 Conclusion

The immune microenvironment after kidney transplantation has important research value and applications for patient survival. This study utilized the CiteSpace software to evaluate potential collaborators and collaborating institutions, status, and cutting-edge new ideas, thus providing future research trends for exploring and developing the relevance of the immune microenvironment in survival after kidney transplantation. China and the United States have been the leading countries in the last decade. Many studies have shown that immune cells play an important role in the immune microenvironment of kidney transplantation, providing a new therapeutic direction for immunosuppression after kidney transplantation. Overall, the results of this study provide valuable information for guiding future research.

## Author contributions

C-LH: Writing – original draft. X-YF: Writing – original draft, Writing – review & editing. YF: Writing – original draft. X-KL: Writing – original draft. YS: Writing – review & editing, Writing – original draft. X-LM: Writing – review & editing. S-WL: Writing – review & editing.
